# Analysis of architectures implemented for IIoT

**DOI:** 10.1016/j.heliyon.2023.e12868

**Published:** 2023-01-06

**Authors:** William Oñate, Ricardo Sanz

**Affiliations:** a Centre for Automation and Robotics UPM-CSIC, Universidad Politécnica de Madrid, 28006 Madrid, Spain; b Universidad Politécnica Salesiana, Quito 170146, Ecuador

## Abstract

Several technological blocks are being developed to provide solutions to the requirements necessary for the implementation of industrial IoT. However, this is feasible with the resources offered by the Cloud, such as processing, applications and services. Despite this, there are negative aspects such as bandwidth, Internet service variability, latency, lack of filtering of junk data transmitted to the cloud and security. From another perspective, these situations emerge as challenges that are being studied to meet the needs of this new industrial era, which means that the important contribution of academia, companies and consortiums, are achieving a change of course, by taking advantage of the potential of the Cloud but now from the vicinity or perimeter of a production plant. To achieve this task, some pillars of IoT technology are being used as a basis, such as the designs of Fog Computing Platforms (FCP), Edge Computing (EC) and considering the need for cooperation between IT and operation technologies (IT and OT), with which it is intended to accelerate the paradigm shift that this situation has generated. The objective of this study is to show a systematic literature review (SLR) of recent studies on hierarchical and flat peer-to-peer (P2P) architectures implemented for manufacturing IIoT, analyzing those successes and weaknesses derived from them such as latency, security, computing methodologies, virtualization architectures, Fog Computing (FC) in Manufacturing Execution Systems (MES), Quality of Service (QoS) and connectivity, with the aim of motivating possible research points when implementing IIoT with these new technologies.

## 1 Introduction

It is well known that the path marked for the development of sustainable systems in all fields of science is de facto, with which, several disruptive technologies are already immersed in this new challenge and others are experiencing a transition in its evolution. In this sense, the field of information digitization is considered a model for the design of field networks in architectures that are being studied and implemented in smart cities, manufacturing industries, recreational services, transportation, and health, among others [ [1,2]].

Within the specific applications mentioned above, the manufacturing sector is somewhat distant from digital infrastructures because the adoption of I4.0 is taking place gradually, with few companies leaping to a digital era for shop floor management (SFM) solutions [3]. The challenges for the manufacturer are the visualization of dashboards on digital platforms and their Key Performance Indicator (KPI), the necessary economic transformation, the susceptibility of IT systems in security, incomplete data packages during real-time transmission, the advancement of new technologies, and specialized human talent (skills and abilities) [4]. The latter challenge is considered as big and urgent by several world-leading companies that have already implemented I4.0 as a smart factory. In addition, these companies mention the importance of implementing new education and academic training programs to meet new professional demands (e.g., IT solution architecture, user interface design, robot coordination), to have qualified personnel to meet the new challenges presented by the new industrial era [5].

Focusing on the new era of industrialization, according to Ref. [6], it is necessary to have adequate and necessary resources to meet the demanding and highly complex parameters demanded by variable production systems, for which [7], asserts that these systems must be flexible, modular and interoperable to handle small batch orders, customized products, change in the supply and value chain, and interruption of processes to save energy. Situations that involve a shift from automated systems to self-optimizing systems, thus [8], describes some priority mechanisms that should be implemented in a manufacturing Industry 4.0 (I4.0) such as the reduction of production and delivery times, intelligent arrangement of tools in the process machine, virtual value chain for quality management and, above all, shortening production processes. In this way, it will be possible to have logical and decentralized distributed applications, typical of a competitive industry [1,9]. For these changes, studies on the storage and management of information from the ecosphere of multiple services offered by the Cloud (storage, processing, memory and bandwidth) have been proposed as an instance [10]. Despite this, the cloud shows communication delays and saturated networks due to a lack of bandwidth, which is due to the information overload caused by the scaling of Internet of Things devices (IoT) [11]. Negative events that have public and private sector groups (business and academia) in the slogan of facing several challenges in the development of architectures and algorithms to achieve intelligent manufacturing, without neglecting the potential offered by the layers of cloud services in their various resources but on a centralized and homogeneous environment, of which [12,13], mention the importance of Cloud service provisioning towards industry proximity, to guarantee resource management and orchestration of component applications and software engines, within decentralized and heterogeneous environments, it is here where Fog FC and EC systems [14,15], aim to solve the challenges involved in Industrial IoT (IIoT) in aspects such as latency, network traffic, energy efficiency, communications and artificial intelligence.

With these new solution paths, storage limitations and computational capacities are mentioned by Ref. [16], who highlights the strengths and weaknesses of near-edge and cloud computing, leading to the hybridization of FC/Cloud services [17], EC/Cloud services, EC in combination with software-defined networking (SDN), network functions virtualization (NFV) and 5G networks [18]. Technological situations aimed at designing sustainable infrastructures for IIoT, capable of offering guarantees to users and their suppliers.

The paper presents an SLR of near-floor computing, modular production systems (MPS), job shop or shopfloor, going through a taxonomy of the FC architectural framework model, highlighting the systems and their functionalities used and inquiring about their drawbacks, the security of industrial control systems (ICS) and the challenges of related topics. The technical words used in this paper are represented in abbreviations, as shown in TableI.

**Table I:** Abbreviations used in this document.

Abbreviation	Technical term
SLR	Systematic Literature Review
FC	Fog Computation
EC	Edge Computing
FCP	Fog Computing Platforms
MACC	Mobile Cloud Computing
FL	Fog layer
EL	Edge layer
EF	Final devices
NF	Node Fog
FNM	Fog Node Machine
HFN	Hypervisor Fog Node
NM	Node Mist
IT	Information Technology
OT	Operational Technology
CL	Cloud Layer
IASC	Industrial Automation and Control Systems
SFM	Shop floor Management
DSFM	Digital Shop floor Management
MES	Manufacturing Execution System
SCADA	Supervisory control and data acquisition
ERP	Enterprise Resource Planning
SAP	Systems Applications and Products in Data Processing.
CPS	Cyber-Physical System
MPS	Modular Production Systems
ICS	Industrial Control System
SDN	Software Defined Network
NFV	Network Function Virtualization
P2P	Peer to Peer network
OPC-UA	OLE for Process Control - Unified Architecture
TSN	Time-Sensitive Networking
QoS	Quality of Service

The paper is organized as follows: Section 2 shows key concepts of this research work; Section 3 describes the methodology; Section 4 presents a review of design taxonomies for IoT architectures; Section 5 shows an architectural model framework for FC; Section 6 presents industrial sector implementations of IoT and IIoT; Section 7 shows MES as part of a smart factory; Section 8 presents the vulnerabilities that ICS can present; Section 9 the discussion; and finally, Section 10 presents the conclusions.

## 2 Theoretical background

Due to the transition processes the industries are going through and consequently to the emergence of different types of computing, some definitions are generalized, poorly explained and misinterpreted; such is the case of [19], who cites in the second chapter of his article some of these inconsistencies. Thus, the following are some interpretations of the literature to define the computations mentioned in this document.

**Fig. 1:**
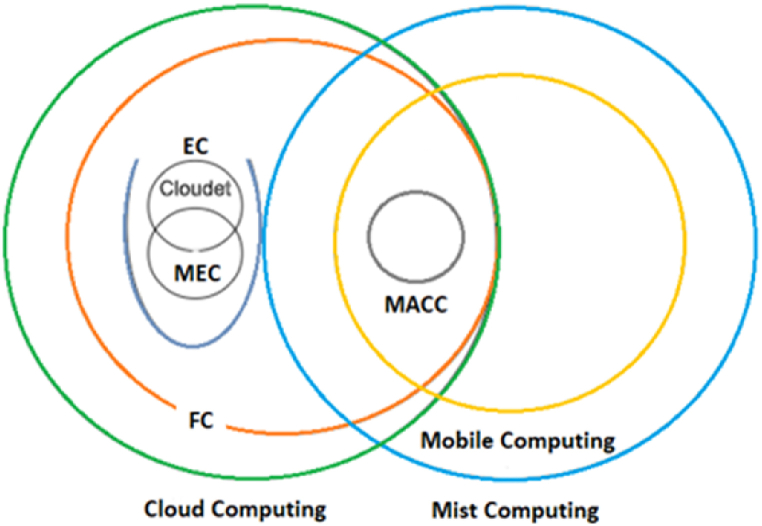
Fields of interaction between computer architectures (Adapted from Ref. [21]).
• **Edge Computing.** As an introduction to the concept of this type of computation, according to Ref. [25], it is designed to lower bandwidth costs, reduce communication latency, and improve connectivity and privacy [19]. mentions that in its beginnings it was used as a complement to Content Delivery Networks and that according to the evolution of its computing competencies (software for virtualization, containers and management tools) it is already considered as a standard for distributed networks, which has allowed it to be coupled in software-defined networks (SDN), virtual networks that operate within physical networks and consequently network functions virtualization (NFV). In addition, EC is known to be isolated from the cloud, but considering its ample computational power and a hybrid model of peer-to-peer architecture and cloud computing would enhance MACC's capabilities according to Ref. [26].

• **Fog Computing.** The good acceptance of IoT, the industrial competitiveness of the evolutionary economy, the longevity of IASCs and the problems that IoT has presented so far focused on industry, are factors that give way to a computation that according to Ref. [20] FC is "A horizontal, system-level architecture that distributes computing, storage, control and networking functions closer to the users along a cloud-to-thing continuum". FC is decentralized, hierarchical computing, which according to its implementation or simulation application, the number of layers hosted in this computing can vary, changing the topology of its network [21]. This computing is located near the edge of the manufacturing process to opt for computing and storage resources, to operate systematically in IIoT systems and therefore CPS (autonomous systems operating in real-time) [22] and is designed to adopt features that allow multiple operations such as the incorporation of other members or nodes to the architecture (scalability-modularity), a generalized computation (middleware) that provides network security services, metadata management for the identification of end devices, interoperability of cloud services, intelligent storage, and all these operations that are accompanied by data routing.
• **Mist Computing.** This computing is also called extreme edge and is located in the first FC layer [23]. With its resources, it greatly supports Mobile Cloud Computing (MACC) by pre-processing them, through Multiple Access Edge Computing (MEC), processing large amounts of geospatial data and then sending them to a cloud database through the fog nodes (NF) belonging to FC [24], as shown in Fig. 1.

## 3 Methodology

The document is intended to discuss those case studies that design architectures and were implemented in collaboration with the digital transformation of the new industrial era, so the literature was evaluated based on criteria such as computations used and implemented for Industrial digital transformation, industrial manufacturing or modular processes in IoT and IIoT ecosystems, digital scaling faced by traditional IASC systems and current systems that constitute a smart factory, however, before these criteria, it is put into consideration those studies simulated or emulated by IT specialists regarding methodologies and architectural designs for FC, which are far from the projection that this document intends to give in cases of implementation, but they are considered as evidence of current and future requirement to have specialists with characteristics in these two branches of IT and OT, to speed up the journey of this digital industrial manufacturing technological transition.

In this context, a systematic review of a total of 119 publications cited by the research community spanning from 2017 to 2022 was conducted, which were analyzed individually in case studies, and collectively through IoT taxonomies referring to industrial processes, to generate an analytical basis for future implementations of IIoT infrastructures. An example of such implementations are the importance of having resilient architectures (fault-tolerant systems), advanced-robust intrusion detection devices (IDS), interoperability and security criteria within all layers that may constitute an infrastructure, and the effect of the number of devices (endpoints and/or nodes) in the network architecture, standards and protocols of communication architectures, cost analysis in terms of scaling IASCs towards digital transformation (DT), correct interpretation of definitions and conceptualizations that DT encompasses, attributes that characterize the devices that make up an IIoT, and certain assessment factors in an IoT communication.

On the other hand, individual works with implementation character focused on the digital factory are also analyzed, for which, it was separated into two parts: The first one extracts those articles that define the pillars of an FCP, the parts of the design that is constituted a framework model for FC as devices, systems, functionalities, service layers, resources and levels. With this base, different optimization protocols are shown for simulated applications for IoT and emulated for IIoT, from which technological bricks and enabling technologies are established in a general way to give continuity to the manufacturing digitalization process, such as layers and tiers as the basis of architectures designs for FC, integration of different types of computations, additional layers within a tier to alleviate processing load in different types of computations, integration and coupling of different types of computations to give IIoT post, necessary IT/OT synergy to assemble an MES under ISA standards, cloud manufacturing/IIOT/IA to give way to MES and MES as a fundamental part to form a manufacturing smart factory. These technological contributions are intended to provide solutions to various parameters such as latency, security, QoS, communication, storage and energy consumption and represent concerns and challenges that will be considered as a description framework in the design of model architectures implemented with the analytics required in the new industrial era.

From the above mentioned, the second part of the individual articles intends to give focus on the analysis of industrial processes under the criteria of requirements for an advanced manufacturing Industry I4. 0, such as manufacturing in the cloud, Artificial Intelligence and IoT with added value or IIoT. All this situation gives rise to MES, where the characteristics of these systems must be contained in smart factories to improve their response capabilities to external stimuli. Therefore, the study analyzed the components that constitute an IIoT in terms of its technology and the way it is applied, such as the network and its intelligent components, CPS systems for autonomous work between machines, associated generic IT and optional computing platforms in the cloud or at the edge, analysis, collection and exchange of information in real-time and intelligent features that allow monitoring of cyber-attacks, because, in the last two, the vulnerability of ICSs have increased. Consequently, publications that worked with IIoT systems implemented with computing from the proximity of a shopfloor, processes, job shop or practical laboratories are shown, which present different methodological proposals on architectural designs based on criticality (virtualization architectures), research proposals for improvement, challenges posed during the study process, QoS, next-generation connectivity and the security they imply. In this way, the intention is to give a holistic look at those points of interest that are being investigated, implemented and put into experimentation, to have a research niche that is of interest to the reader and help to face the changes in the industries that are going through.

This research was developed in high-quality bibliographic database platforms such as Science Direct, Scopus, Institute of Electrical and Electronics Engineers (IEEE), SpringerLink, Google Scholar, ResearchGate, white papers and digital spaces of consortiums for the demystification of publications.

The search of the publications was through several topically essential keywords, which could be grouped or individual according to the search need, to mention a few examples: ["Fog Computing"], ["Mist Computing"], ["Edge Computing"], ["SDN/FVN"], ["Optimization"], ["Taxonomy"] ["Service Classes"], ["Resource Performance"], ["Fog Computing Platform"], ["Model Framework for Fog Computing"], ["IIoT"], ["MES"], ["Virtualization"], ["TSN"], ["OPC-UA"], ["5G"], ["Blockchain"].

## 4 Partial taxonomy

IoT systems are presenting adaptation characteristics in IASC, due to their greater openness, scalability, heterogeneity and dynamism. However, there are challenges that must be addressed from different points of view, which require a review of taxonomies of IoT architectures developed so far, considering that there is no specific architecture as such and consequently this article aims to leave as a basis an analytical for the implementation of future IIoT architectures, of which [27], in its systematic review, classifies IoT applications in the following lines of research as smart cities, health care, commercial, environment and industry, the latter being the one that reaches only 10% and its studies are mainly directed toward latency factors with a focus on programming applying IT and that of this percentage approximately ¾ of the reviews focus on simulations and designs (neither implemented nor simulated), and that approximately one-third of the reviews focus on simulations and designs (neither implemented nor simulated) ¼ are truly implemented, consequently, in view of the evidence of a delay in the evolution of IoT applications for the industrial sector. With this background, certain studies with relevant information were located to venture into relationship issues, which [28] emphasizes that IoT systems must operate within a domain of resilience, regardless of the number of layers it is made up of. This implies an emphasis on privacy and identity management mechanisms, with the practical aim of having fault-tolerant processes, through intrusion detection devices (IDD), physical protections and connectivity interruptions, from plants, nodes and clouds, for which the authors mention the complexity that IT requires for central control at each layer, between layers (demilitarized zones) and between all layers. In this way [29], its systematic review starts from a natural heterogeneous vision of the IoT and its vertiginous evolution towards large industrial systems, for which it makes a classification of the types of malicious activities that affect the IT ecosystem of the IoT, deepening and detailing the classification of the subcategories of contemporary AI involved: Signature-based Intrusion Detection System (SIDS) and Anomaly-based Intrusion Detection System (AIDS) and, within its taxonomy, the authors mention that an advanced IDS, must be constituted by 4 fundamental elements for its development: (1) evasion of apparent alarms depending on the volume of data, (2) adaptation to communication systems at the process level (the unexpected action of sensors), (3) detection of pre-analyzed attacks and new vulnerabilities from day zero, and (4) implementation of autonomous IDS for volume of data.

One IoT contribution to interoperability and security is the application of the 6LoWPAN standard for the transmission and reception of data between devices with static IP addresses (V4 or V6) through the IEEE 802.15.4 communication protocol, that is, those devices that meet the parameters of connectivity, energy, processor, storage, security, reliability and detection can be part of an IoT infrastructure. However, the integration of an IPv6 packet in an 802.15.4 frame leads to an increase in latency and resource consumption (CPU and RAM), which is extremely important to consider when leaping IIoT, when there is a significant range of volume of end devices that cannot be supported with the addresses offered by IPV4 [30]. In this way [31], in its comprehensive security survey for a four-layer IIoT architecture (device layer for identification of things (physical and virtual), network and transport layer for protection of end-to-end communication across networks (backbone, backhaul, capillary), processing layer for protection of end-to-end data in a state of (rest, The application layer for communication architectures (request/response or publisher/subscriber), states that the problem is interoperability due to the heterogeneous volume of devices and processing and processing nodes respectively, a situation that arises due to the complexity of identifying different addresses, data frames, structures and security protocols according to network requirements. These situations require more powerful processing equipment than those used by traditional IASCs, where the process is centralized, proprietary equipment and a finite number of devices, from which even the security systems are different from the new ones proposed for IIoT, scenarios that leave a bittersweet taste in terms of the change of era and the high cost of scaling.

On the other hand, the work of [32] deepens in several definitions of digital transformation applied in IASC such as I4.0, CPS, Industrial Internet, IoT, Supervisory control and data acquisition (SCADA), Distributed Control System (DCS) and IIoT, and from their point of view they are poorly conceptualized and interpreted by different researches. In addition, the authors intend to differentiate IoT and IIoT architectures based on the characterization and way of acting of devices in a branched tree structure typical of IIoT, for which they perform a taxonomy of research on those attributes that devices belonging to an IIoT system must have, such as type of industrial sector, device location, connectivity, device characteristics, device technology and type of user.

The following are the communications that are used in IoT, analyzing the lines of research that these are subdivided, so that the resource and intrusion-based communications, presented by Ref. [33], represent a higher percentage of research papers compared to routing and monitoring, the latter being of importance in industrial scenarios such as in SCADA Web or IIoT systems for device-to-gateway (D2G) and device-to-cloud (D2C) communications. Regarding communication technologies, there is no significant contribution due to the generality addressed in its analysis and SLR results and it does not deepen in existing communication protocols and their advances. Despite this, it makes known those valuation factors for IoT communication that are being applied by researchers, such as response time, computation time, execution time, bandwidth, power consumption and latency demand. Moreover, the percentages of less impact but not less important are delay, utilization (network, batteries, CPU), security, performance, scalability, availability and costs.

## 5 Architectural framework model for Fog Computing

**Fig. 2:**
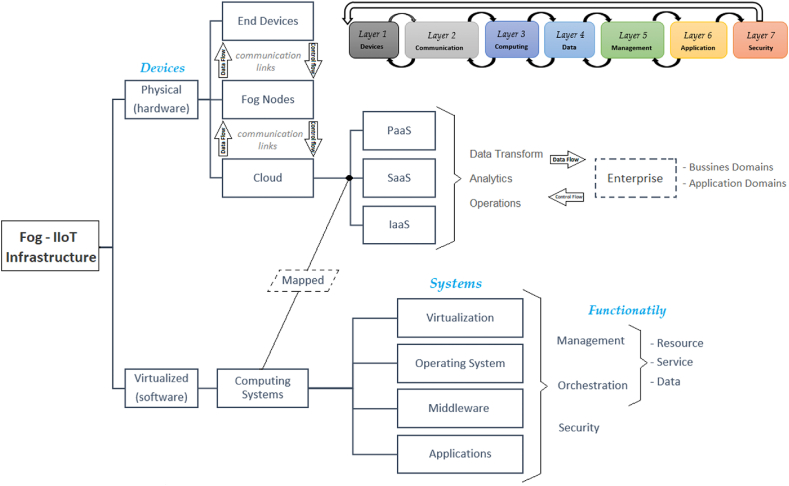
Levels of a modeling framework for fog computing (adapted from Refs. [35,36]).

From the above mentioned, there are currently several computing technologies used as control mechanisms for cyber-physical systems (CPS) relating the physical and digital parts of an IIoT system, considered this system as a fundamental pillar for manufacturing processes. With this approach and in general terms, the levels that constitute a framework for CF are presented according to the physical devices, computing systems and their functionalities, as shown in Fig. 2. In the same vein [34], highlights the overlapping aspects between IIoT and IoT in their development and the differences between them, such as service models, criticality, connectivity, data volume and QoS. Previous factors to consider in the design of the functional model of an IIoT architecture.

**Fig. 3:**
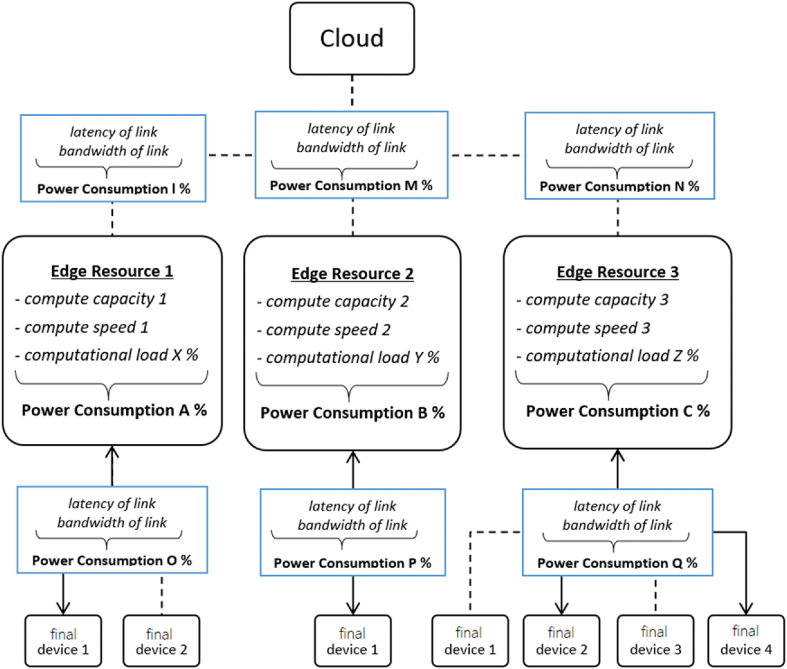
Resources for a three-layer HF model framework (Adapted from Ref. [37]).

It can be said in a non-trivial way that IIoT requires for its development and implementation several capabilities such as computing, storage, communication networks and cloud services, which are necessary to have them or direct them to the edge of the process to support the end devices (ED) of a manufacturing process, from which [37], rightly mentions that the functionality of the resources must be optimally allocated to increase the performance of the system [38] (Fig. 3). The optimized quantifiable study resources are processing capacity and memory per workload [ [39–41]], consumable bandwidth between PEs with computer system nodes [42], routing protocols [43] and cloud services mapping [44].

The innate scenario of infrastructure for IIoT solutions highlights the importance of efficient energy consumption, due to the fixed and constant economic costs over time. In this regard [45], states in its document, that no matter what virtualization architecture is designed, the energy drawback is present, a situation in which [46] proposes the development of a machine learning (ML) model and standardized evaluation metrics for the management of quantifiable resources such as energy through load prediction, but also adds that the existence of non-quantifiable quality attributes of QoS for FC such as security, mobility, availability and reliability should not be overlooked [47], requirements that, according to the documents presented, point out as future work of interest, the need to optimize those metrics that allow measuring this type of requirements for QoS.

Other authors highlight the design of architectures for FC as an open architectural framework due to the ability to enable Fog-Cloud and Mist-Fog interfaces. Thus, on an SLR of several architectural studies for Fog, they take as a reference point for their designs the FCP for IoT, such as the European Telecommunications Standards Institute (ETSI) for standardization in the field of FC and EC and the OpenFog consortium based on the IEEE 1934–2018 standard, in which [20,48] clearly describe the attributes that an FC architecture should possess, designed to address performance, latency and efficiency issues addressing networking, storage, control and acceleration; despite all this information, in chapter 3 of [48] show several practical use cases using this type of computing platform, but there are no studies in industrial manufacturing processes. Despite these limitations, the authors in Refs. [49,50] perform a holistic analysis for the scaling of IASC in digital technology, from which they identify and suggest possible solutions in industrial use cases for automation, robotic cells or mobile robotics, concluding that the above mentioned would be possible if there is integration between IT and OT, where IT consolidates the necessary pillars for each hardware and software component to operate as a single whole providing cloud and OT services for the identification of those physical components that constitute the production process and its control.

## 6 Industrial sector implementations in IoT and IIoT

### 6.1 Simulated implementations

A turbulent and interesting scenario that results in numerous studies that aim to provide a general or specific solution according to the level, field or sub-field of the CF framework model. However, most of the papers found in the study mapping are hypothetical and few are implemented, using as a case method software and laboratories of virtual environments for IoT; a scenario that is far from real applications for industrial processes, slowing its acceptance and further marking the emerging paradigm that is going through the computing near the edge [51].

Despite this information, studies that were simulated and/or emulated for CF are presented below, from which there are several proposals on the improvement of infrastructures in dynamic resource provisioning [52] developing different methodologies that operate close to a shopfloor (Fog or Edge architecture). In this sense [53], proposes an FC framework called FITOR, through orchestration of resources for devices of different layers, services and links, adding value to Service Deployer for the optimization of location, computation and network requirements in Fog nodes (FN) and Mist nodes (MN) [54,55]. The author in Ref. [56] extends a framework for FC called ENORM, where there is a service management administration between cloud servers and partitioned servers on the Edge node [57]. proposes a framework design for FC called Foggy, supporting large-scale geo-distributed IoT applications in a general-purpose three-layer infrastructure (Edge, Fog and Cloud), its contribution is the automation of the distribution of specific codes for low-scale nodes, requested by users. Despite this, the implementation presented is not performed on a large scale as presented in its design [11]. It presents a cloud orchestration framework that allows the modeling of automated applications and services, through an IoT-PaaS architecture based on NFV for a hybrid Fog/Cloud system. Its plus is in the automation of an architecture for the provisioning of IoT applications on Cloud and Fog resources [58]. uses a simulation environment called iFogSim, which uses six temperature sensors, the implementation of an algorithm for the identification of the previously defined bandwidth excess and a Gateway-Cloud link [59]. proposes an efficient indexing model for the Fog layer in IIoT, in a theoretical and experimental way. The latter uses a functional test simulation platform for web services but does not specify the kind of services that may be needed in the edge data centers, the needs required in the production plants, the monitored data or control information. Leaving a doubt on the need for the use of WSDL/SOAP or REST based services.

It should also be considered that during the pandemic period, there were studies of positive reviews and simulations in virtual laboratories with topics related to methodologies for Fog, hybrid Fog/Cloud and Edge/Cloud computing, implemented (simulation and/or emulation) in the Industry, but without real laboratories or equipment, for the consolidation of knowledge acquired through the interaction between theory, experience and learning [ [60,61]]. All this research information is of great interest, but it should be considered that real implementations will be feasible in the short and medium term. This situation leaves an intriguing panorama on the dark side of the IIoT since the integration of IT and OT is necessary for its evolution. In this regard [62], define ‘‘IT/OT convergence as the integration of IT systems applied to data-centric computing with OT systems used to monitor events, processes, and devices and make adjustments in enterprise and industrial operations. IT is composed of those hardware and software system technologies that allow for corresponding information processing. OT is supported by physical devices, that is, switches, sensors, power distribution networks, valves, motors, and software that allow for control and monitoring of a plant and its associated equipment’‘. With this background and starting from IoT, several studies emphasize IT, simulating its architecture and others emulating OT scenarios, which lack a reality at the process level of the manufacturing zone of the Purdue model, a situation that adds the delay in research and implementations of IIoT ecosystems [63], which represents a current situation because the IT/OT synergy is empirical, as a result of the separation of these two technologies from the academy. Thus, to give continuity to the construction of people, processes and technology, there are some higher education institutions in different developed and emerging countries that offer study programs with technological trends as a contribution to the digital transformation [64–66]. All this collaboration will allow industrial automation for real systems. Despite this reality, there are other criteria to consider for industrial implementations such as those specified in the methodology and also other variables such as costs, lack of research availability and the importance of not generating ambiguities of conceptualization, taxonomy and interpretation of architectural frameworks, devices that make it up, computations, communication networks and/or service models [67].

### 6.2 Case study implementations

#### 6.2.1 Hierarchical architectures

Consequently, this section intends to investigate the architectures implemented in systems such as MPS, shopfloor, laboratory processes or job shop, such is the case of a design for digital shop floor management (DSFM) [68] through a framework for vertical and horizontal communication between two manufacturing processes, each framework consists of 4 layers in its architecture (Edge/Machinery-Office, Mist, Fog and Cloud), its communication through push technology and a centralized node for enterprise resource planning (ERP). However, in the actual implementation, only three layers are implemented (Edge/Machinery, Mist and SAP Cloud), where the author specifies that the Fog layer for the provisioning of ERP enterprise resources from the SAP S/4 business suite from a SAP-Cloud services platform will be arranged in other future research and that the only activity performed by the Fog layer is that of Gateway and not Middleware, so its design is different from the one implemented.

[69] develops a vertical communication framework for secure data storage and IIoT search through Fog/Cloud computing integration, due to the instability presented by the EFs such as low quality of transmitted data, false and missing values, motivated to develop a transformation of data into a unified resource description framework and then merges them for the elimination of redundant data, improving the efficiency of data storage already existing in the FL and Cloud, consequently releasing stress on the network. Its architecture presents a 4-layer design (ZigBee sensor network, Edge/server, Fog/perimeter proxy server and Cloud/server), achieving greater data security in the perimeter servers (proxy and cloud). Despite this, the provisioning of cloud services does not apply in the LF and the implementation is executed in a laboratory through the reading of six temperature sensors, features that do not represent a real system of processes of an aggregated automatic smart factory, shopfloor or job shop factories for automation.

On the other hand [70], proposes a method of scheduling and energy-aware load balancing (ELBS) by robots and using NF. Its architecture is constituted of three layers (Mist/sensors-actuators, Fog/Switch and Cloud/server), where the Mist layer (ML) plays an important role in the development of a dynamic scheduling system based on multi-agent technology and PSO-enhanced particle swarm optimization, to achieve a multi-task and multi-object cooperative control necessary for the energy consumption model in the ML nodes (agents). The communication protocol that supports this system is a dedicated network and the implementation is developed in a job shop (candy container) with similar characteristics to an industrial process, consisting of five workstations with robotic arms and a conveyor belt that communicates the processes, however, the central node belonging to the LF subsystem operates as Gateway and does not perform any provisioning from the cloud, making clear the non-use of any software as middleware.

[71] propose to perform an augmented reality (AR) application applied to the shipbuilding industry, considering that the aim is to migrate to a current architecture where the content of the I4. 0 becomes dynamic and demanding, with higher video bit rates and lower latency demand. That means, if a local cache is used for the storage of compressed video frames, the complexity involved in the effectiveness of prediction algorithms and the constant updating of data, all this suffers a significant increase in processing power costs, so the authors implement three architectures for image processing based on Cloud and FC, the latter being an architecture composed of three layers. In the first one, clients sent concurrently simulated digital display information (IAR), and the second one is called LF, which is constituted by several computers on a single Orange Pi board, cooperating as Gateway, raw file, and multimedia object detection for the intensive calculation of the photorealistic image generation process through AR services, and the LC is connected with third parties such as SAP to grant ERP services, MES, Windchill software for product lifecycle management (PLM), FORAN as CAD/CAM/CAE system of the 3D models and the ThingWorx platform for industrial IoT. Despite the information presented, the authors do not specify if the LF cooperation nodes were orchestrated, or failing that if it used any kind of switch that operates with NFs, or if it performed any kind of provisioning of the service or application resources for AR from Cloud. Regarding its results, it leaves a proper look at the behavior of the three architectures in terms of load variability, latency and data transfer rate.

**Fig. 4:**
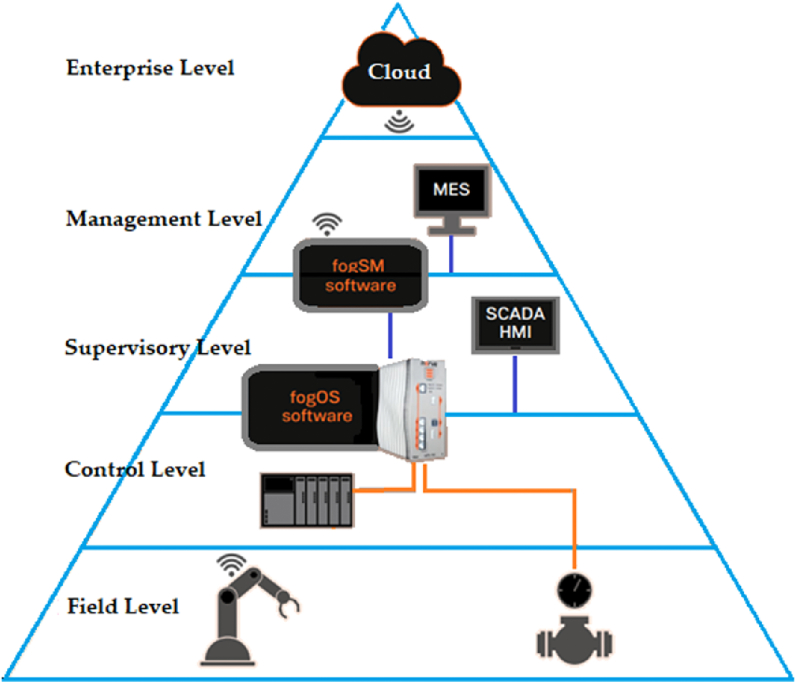
TTTech Industrial's commercial hierarchical architecture (Adapted from Ref. [72]).

So far, several studies show the weaknesses in hardware and software, but some companies take the risk and go a step further by launching devices called Fog Node Machines (FNM), which are made up of specialized software and hardware for IIoT solutions. These FNMs operate on a software platform (FogOS), composed of multi-chip processors (MCP) for hosting applications and virtual machines (VM) through different types of hypervisors for static or dynamic work (ACRN, Xen, KVM, PikeOS, etc), depending on the requirement of the level of operation within its architecture, as shown in Fig. 4, a field level for processing, storage, analysis and visualization of information, up to the level of supervision, factory or enterprise.

The FogOS platform's FNMs have the standard plant level Fieldbus for connectivity and incorporate time-sensitive networking (TSN) switch under the unified architecture of OPC (OPC-UA) and deterministic Ethernet with open standards. In contrast, to achieve connectivity between the NF of the FogSM platform to the cloud provider, it implements the TCP/IP model and a lightweight MQTT protocol. However, their costs are high [73] and their implementations so far are considered pilot projects [74].

Despite the information of IIoT systems implemented in Job shop, it should be mentioned that the information regarding use cases in renowned industries such as oil, gas, transportation, water management, utilities or others, is deficient or almost null. This is because these companies have policies not to share or disclose information details such as standards, security and connectivity, considering other companies as rivals [75].

#### 6.2.2 Hierarchical architectures

**Fig. 5:**
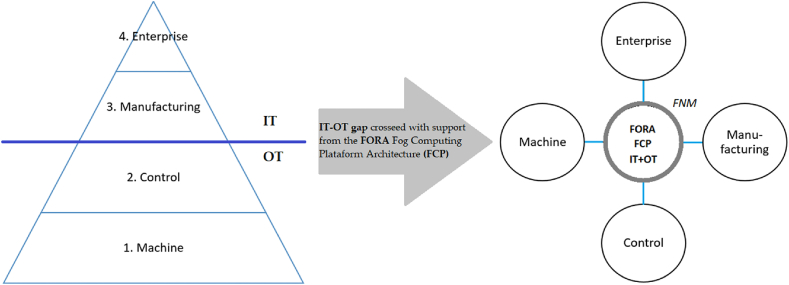
From a hierarchical architecture to a flat architecture (Adapted from Ref. [82]).

Taking as starting points, pillars for FCP and standards already defined for IoT [76], develops a FCP with several technological challenges for a futuristic architectural design capable of interacting with distributed CPS, to provide a low latency demand, through different requirements such as virtualization at the operating system level or partitioned architecture through VM for the execution of applications or subsystems with mixed-criticality in isolation, time, space, storage and fault identification and correction [77], develop specialized middleware for the exchange of information between applications contained in a VM or between VMs, specialized hardware to support different kinds of containers and hypervisors, security against cyber-attacks at the Fog level through network security policies [78], resource provisioning with priority given to low-latency demand [79], orchestration and resource management [80] and state-of-the-art adaptive connectivity TSN [81]. Current requirements for the transformation of a traditional automation architecture with a hierarchical inter-tier communication methodology to an FCP with a P2P communication methodology through an FNM, as shown in Fig. 5.

Thus [82], proposes a method for the use of computing resources close to the production plant and with a fully distributed P2P communication functional and evaluated for three industrial use cases. To achieve its purpose and the complexity required by the system to be built, an Architecture Analysis Design Language (AADL) and a guide as a methodology proposed by the European Training Network (ETN) for FC in Robotics and Industrial Automation (FORA) were used [83]. Its first industrial use case is the start and position control of 5 motors of a conveyor belt and the labeling of products, where [84] develops an FCP under AADL to model what would be an NF architecture, identifying bottlenecks and requirements that are needed as technological bricks for FC [85]. concludes his research by assuming that all applications and data signals are transmitted at a speed of 100 Mbps and no delay is observed regarding the arrival of information to the different applications or inputs, respecting flows, criticality and without temporal variability, fluctuations evaluated by Jitter Time.

**Fig. 6:**
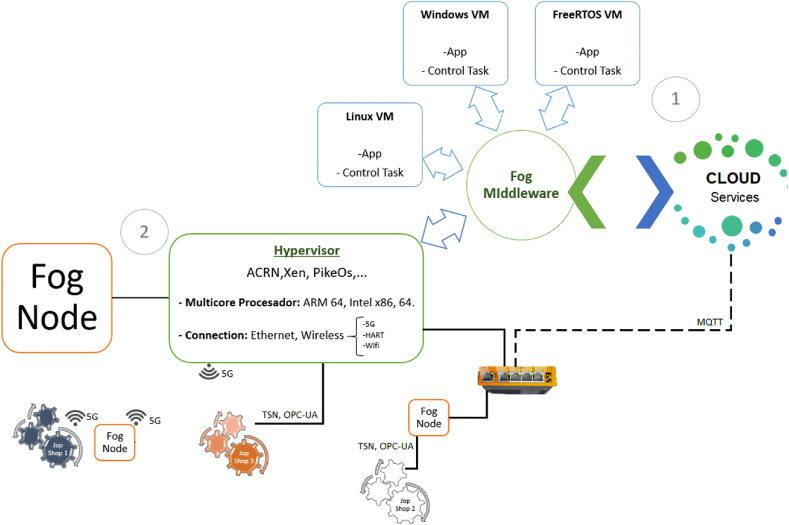
Flat architecture with HFN for Shopfloor (Adapted from Ref. [90]).

Despite the research on different fields that comprise the IIoT, several authors mention that there are still issues that need to be improved in order to align with the IEEE1934-2018 Open Fog standards, such is the case of [86], which states that network connectivity for CPS through a standard Ethernet switch for NF (deterministic real-time networks), still has drawbacks in network synchronization, of which [87], mentions that mechanisms with the capacity of fault tolerance through TSN systems are currently being developed at the level of exhaustive simulations, so [88] makes a comparison between two modelers; TSN's own Time Aware Shaper (TAS) and Asynchronous Traffic Shaper (ATS) [89], on a network that emulates occasional randomly perturbed traffic characteristics suitable for ICS, other researchers delving even deeper such as [90], which mentions the importance in the unification and estimation provided by TSN and OPC-UA technologies, comparing the TAS and Credit-Based Shaping (CBS) modeling of TSN, when faced with a read request from the OPC-UA server, with high priority 7 window conditions and disturbances of up to 100 Mbps. The integration of the above scenarios is illustrated in Fig. 6, a possible design for an FCP according to FORA's intended requirements for a Hypervisor Fog Node (HFN).

The architecture is divided into two parts. The first one, arranged for communication between three Job Shop stations towards an HFN, where workstation 1, uses as communication technology TSN wireless known as wifi 7 or 5G under IEEE 802 and 3GPP standards, workstation 2 uses for its connectivity TSN running on an Ethernet bus and workstation 3 with an OPC-AU communication protocol over TSN, through a TSN switch for real-time converged networks, this switch acts as an architecture towards the cloud, that is the FCP can handle mixed wired and wireless TSN connections through the hybridization of two protocols TDMA (Time Division Multiple Access) and NOMA (Non-Orthogonal Multiple Access). In the second stage, the HFN is equipped with a multi-chip processor (MCP) to support containers or VM virtualization, through a hypervisor intermediary to provide: real-time operating systems (RTOS), flexibility and concurrent execution of applications. On the other hand, the HFN also incorporates ISO-MQTT and TCP/IP standards for communication to the Cloud. In addition [91], specifies the performance benefits of a model with P2P architecture, suitable for bandwidth reduction and consequently the latency for communication between the HFNs of the Edge layer (EL) and the possibility of developing mechanisms for fault tolerance, which is not the case with the hierarchical model. However, the Linux Foundation [92] mentions textually that "Virtualization can help meet these broad needs, but existing options do not offer the right combination of size, flexibility and functionality for IoT development". Furthermore, this statement has a focus on IoT systems, consequently, the drawbacks and challenges raised in this paper when it comes to IIoT systems must be added to these.

## 7 Manufacturing Execution Systems (MES)

Knowing beforehand that the new industrial era 4.0 is a decentralized system and requires its purpose to have an intelligent factory, then it would be talking about CPS systems, where its main manufacturing features are autonomy and real execution time between these systems, machines and/or humans, also remembering that the IASC to be considered only as OT and being relegated to a local network (in the best of chaos) and without IT, a clear example according to Ref. [29], would be the SCADA systems because it has internet connection but its connectivity is limited and also lacks data analytics, consequently the author recognizes SCADA as the predecessor of IIoT. Consequently, for an IASC to have the qualities of a smart factory, it is necessary to first analyze the parameters of an IoT applied to the industry such as availability, latency, energy consumption, cost and reliability [27]. As a result, advanced industrial process control systems I4.0, according to Ref. [93] MES is a key factor for manufacturing businesses to become smart, whereby the incorporation of IIoT, ML, and cloud manufacturing (CM), are direct aspects that increase responsiveness and become responsive to the customer. To achieve the aforementioned challenges. The author in Ref. [94] specifies that the main pillars of MES are: decentralization, vertical integration, Connectivity and mobile, and Cloud computing and Advanced Analysis. Additionally [95], in its exploratory review on the security of MES systems under Quality function deployment (QFD), mentions that IT/OT technologies are established in the ISA 95 standard, as explained by the following studies.

In [96], the authors propose a solution for automation aimed at SMEs with smart factories, model-driven in terms of a hierarchical architecture with a scalable and flexible approach, and a data model applied to the collaborative work of robots for the assembly of parts at the request of different orders by the customer. For this action, the authors use the ISA95 standard as a guide and propose a data flow from the business layer to the production teams, using an Odoo open source enterprise platform for integrated ERP services, with PostgreSQL database, which allows establishing an interaction between the business and MES layers, The latter allows interaction between the MES and control layers through an API such as MySql API, the latter communicating with the device layer (client-server) through the Open Platform Communications and Unified Architecture OPC-AU protocol [97]. The presentation of the case study implemented in this article is shown in a general way, where the architecture designs and data models are shown as a guide for the design of the MES system, but it does not go deeper into topics such as algorithms, Oddo platform services, resource management for load distribution among the robots of the production layer, methods and/or metrics to evaluate its results.

With a more ambitious approach [98], proposes in this case to improve the previous experience, incorporating an MES/manufacturing operations management (MON) system to an experimental laboratory smart factory, with the following features: a data model based on ISA 95, IIoT for vertical integration, interoperability and order customization, and quality function deployment (QDF) through a secure distributed architecture. Despite this, the authors after experimentation mention that practical MES designs are costly and difficult to implement, with which, after experimentation, the authors establish three principles for future MES implementation designs: i) flexible reconfigurable system (hardware and software) at all levels of the architecture, regardless of the architecture and regardless of the devices, ii) easy to operate environments for users, from anywhere, at any time and in real-time, in obtaining the information they need and iii) being a CPS system, the need for security systems throughout the system quality function deployment (QFD) is sought, knowing as the first link of a possible attack to the users.

The authors in Ref. [99] design a bidirectional architecture that allows the detection of anomalies in a process (drilling speed and number of finished parts). This architecture consists of an EL with two sub-agents each responsible for monitoring and collecting data from assets 1 and 2 arranged in the device layer. The central agent is located in the MES layer, formed by two repositories, the first one, for the variable behavior models, collaborated by a machine learning (ML) algorithm for the identification of anomalies in the variables of the process assets, and the second one is constituted by topologies, which are assigned according to any inconsistency occurring in the process. However, the author considers the change of topology as the power supply cut to the asset(s) 1 and/or 2, with which, the corresponding sub-agent(s) of each equipment would send notification of what happened to the MES layer. In the same way, as the previous study, this article shows a general content of its entire infrastructure and its results are not quantitative.

## 8 Security in industrial control systems (ICS)

It is known the high potential of processing, applications and services that Cloud Computing can offer to IIoT, despite this, there are several studies such as [100–104], which mention the disadvantages compared to other computations, in factors such as increase of bandwidth and its consequent cost as devices and CPS systems increase in process level, variability in the provision of internet service, problems in latency and synchronization frequency times, lack of filtering of junk data transmitted to cloud and security; the latter being a block of the functional system that should be treated with the importance it deserves, due to the lack of reliability that IT presents to the detection of cyber-attacks, regardless of the type of architectural design that is implemented, adding [105] in its 2020 report, that vulnerabilities to industrial control system (ICS) have increased by 49% since 2019.

Against this background, several studies have emerged, such as the one that [106] externalizes in their work and explains that those systems that handle different communication protocols in their architecture are a target for cyber-attacks, with that approach the authors propose a security model based on machine learning (ML) of the random subspace (RS) and random tree (RT) models towards a SCADA with IIoT network traffic, from another point of view [107]. say that cyber-aggressions fall with the OTs operated from Cloud, this is because the security and privacy of the network are based solely on password authentication, being this method unreliable, as well as [108], who exposes the imperative need in the use of a common vulnerability scoring system (CVSS) for critical, complex and large systems such as SCADA, because they can identify through their metrics the existing vulnerabilities in communication protocols or algorithms, in that way it is timely for taking actions from IT. In the same way [109], points out that these cyber-attacks are also directed toward DEs, of which in their study they propose the optimization of the PAKE protocol "of a verifier-based password-authenticated key-exchange (V-PAKE) protocol as a hedge against public-key-infrastructure (PKI) failures is considered important", achieving higher performance in processing and speed of response to cyber-physical attacks. On the other hand, the transfer of an emerging IoT-Blockchain technology to industrial processes is being accommodated, aiming to improve security both in the access of information and properties coming from the DEs, registering them in a database for later analysis from a server, of which [110], assures after its IIoT implementation in a Cloud architecture, that Blockchain grants a secure connection of DEs. However [111], exposes an important point of view when considering that there is a marked difference in providing security to IIoT and IoT systems based on Blockchain, due to the type and variability of the information and attributes that EDs handle, facilitating the embedding of undetectable false information. In addition, QoS becomes problematic and uncertain to measure, due to the instability in the series of finite transactions (FST) that occur in continuous processes typical of automated manufacturing. These studies are intended to provide security for external and internal cyber-attacks to the ecosystems mentioned in this document [112], improve QoS in communications over the end-to-end connection with 5G networks [113], and ensure industrial wireless connectivity for DEs and nodes close to manufacturing processes [114].

Despite this, considering the existence of several current architectural frameworks for IIoT and that they present different numbers of layers [115], identifies those threats and vulnerabilities present in various 5G-based networks or other similar ones such as Dynamic Network Slicing Orchestration applied towards SDN and NFV for the smart factory [116], radio access network (RAN), non-orthogonal multiple access (NOMA)-based MEC edge system computing for 5g-IIoT [117], SDN, 5G networks applied in SDN-NFV [118]. To cite an example [36], based on a tiered framework, as shown in Fig. 2, but with a generic focus on 5G-IoT networks [119], detects eight serious threats to sensors when using 5G networks in IIoT.

## 9 Discussion

When it comes to industrial applications implemented on IoT systems, section 4 of taxonomies reveals that a small percentage presents this line of research, this is because IoT in its functionality and technology has limitations for the requirements of the new generation industry. Consequently, several case studies are applying value or added value to IoT to now have an industrial IoT system, with features that allow it to operate within a smart factory and cloud services, which means that its functionality and technology can incorporate CPS systems for autonomous work between machines in real-time, operation in networks with different architectures and communication protocols, associated generic IT and optional computing platforms in the cloud or at the edge, IT/OT-MES, intelligent features for monitoring a large amount of data and security throughout the system.

Therefore, implemented IIoT applications are scarce and those found in the literature, for example in DSFM-ERP systems, it is observed that they introduce FC as a solution with virtualization from the operating system for a hierarchical architecture model, however, they are presented as implementations for IIoT, but in the case study they do not meet 100%, leaving FC with a low valuation by not fully exploiting its benefits. On the other hand, the influence of FC in MES systems for IIoT has been observed in other case studies, opening a possibility for the implementation of these systems. Despite this, these documents do not detail aspects such as the standardization of metrics used, algorithms or optimization of these at any point of the model of the architectural framework, security in process level, links, virtualizations or in the cloud during the provisioning of resources, that is, they would seem to be documented on hierarchical architectures designs (sensors/actuators-FC-MES-ERP), being reluctant to disclose their information product of the gain of knowledge in their successes and errors, considering this action as frustrating for other researchers.

Several proposals of virtualization architectures have been observed near the production plant with different Fog, Edge or other hybrid computations, but they are far away as implementation for case studies, meaning that there are several proposals of simulated or emulated industrial character, scenarios of those professionals specialized in IT and others in OT. This leads to a delay in research production due to the need to update knowledge and new learning skills that researchers must acquire to be aware of the necessary synergy between these two technologies to accelerate this current paradigm.

Another point to observe was the development of a flat horizontal P2P architecture for HFN with a hypervisor virtualization architecture; however, it is evident that those consortiums that lead these investigations show promising results in several areas for IIoT solutions such as connectivity, security, provisioning, orchestration and administration. So, starting from the hardware, it is necessary to have a CPU that supports the simultaneous execution of VMs depending on the process requirement and consequently its development and the cost that this equipment represents. As far as IT is concerned, it has been observed inconvenient to have an intelligent system at the edge, capable of operating several VMs with different Operating Systems at the same time, aiming to provide solutions for resource provisioning, data storage, orchestration and task management. On the other hand, the security of having only one password for network authentication or the use of different communication protocols in the same infrastructure, the optimization in the subsets that make up the framework of the architectural model for efficient energy consumption and connectivity due to current standards. All these requirements are undoubtedly typical of intelligent manufacturing of the fourth industrial revolution, but they are still being studied, and there are several research niches and opportunities to be improved and implemented.

## 10 Conclusions

The requirements involved in developing an architecture capable of meeting the demands of IIoT are varied, from research that identifies the difficulties of operating with IoT systems in the industrial sector, studies that simulate industrial systems, and the need for specialized personnel in the management of both IT-OT technologies, consortia that propose the construction of novel platforms with different virtualizations for computing in the vicinity of the production plant and implement them in DSFM, ERP and MES, challenges in hardware and specialized software.

After an SLR, this article aims to show in a general way, those real case studies implemented for IIoT, to know the current enabling technologies and the specific concerns that leave these proposed systems, with which it is intended to generate motivation or concern for future research aimed at real implementations.

## uncited Reference

39 40 65 101 102 103
